# Integrated Analysis of Chromatin Accessibility and Transcriptional Dynamics of the Viral Genome Following Pseudorabies Virus Infection

**DOI:** 10.3390/ijms27167100

**Published:** 2026-08-07

**Authors:** Songbai Yang, Kun Teng, Guijun Liu, Xiangchen Li, Han Wang, Ayong Zhao, Xiaolong Zhou

**Affiliations:** Key Laboratory of Applied Technology on Green-Eco-Healthy Animal Husbandry of Zhejiang Province, College of Animal Science and Technology & College of Veterinary Medicine, Zhejiang A&F University, Hangzhou 311300, China; sbyang@zafu.edu.cn (S.Y.);

**Keywords:** pseudorabies virus, ATAC-seq, RNA-seq, chromatin accessibility, viral genome

## Abstract

Pseudorabies virus (PRV) is an important α-herpesvirus. However, the chromatin state and transcriptional regulatory mechanisms of its genome upon entry into host cell nuclei remain poorly understood. In this study, we employed the Assay for Transposase-Accessible Chromatin using sequencing (ATAC-seq) and RNA sequencing (RNA-seq) to analyze chromatin accessibility and the transcriptome of the PRV genome in PK15 cells at multiple time points post-infection. ATAC-seq analysis revealed that the proportion of viral reads increased progressively over time, from 0.01% at 4 h to approximately 1.09% and 13% at 8 h and 12 h, respectively. A total of 112 ATAC peaks were identified, distributed across the PRV genome without apparent low-accessibility regions. Fragment length analysis demonstrated that the PRV genome does not adopt the regularly phased nucleosome organization typical of the host genome. RNA-seq analysis detected 66 expressed PRV genes, which were classified into four kinetic expression clusters, corresponding to immediate-early/early (IE/E), early (E), early-to-late transitional (E-L), and late (L) gene groups. Virus–host correlation analysis revealed significant associations between PRV gene expression and multiple host inflammation-related genes. Integrated multi-omics analysis further confirmed the concordance between chromatin accessibility dynamics and transcriptional activity changes. Collectively, these findings indicate that the PRV genome maintains a predominantly open and accessible chromatin state throughout lytic infection, providing new insights into the epigenetic regulatory mechanisms of α-herpesviruses.

## 1. Introduction

Pseudorabies virus (PRV), also known as *Suid herpesvirus 1* (SuHV-1), is the causative agent of Aujeszky’s disease in swine. It belongs to the genus *Varicellovirus* within the subfamily Alphaherpesvirinae of the order Herpesvirales [1]. PRV infection causes acute fatal encephalitis in piglets, reproductive failure and abortion in pregnant sows, and respiratory disease in finishing pigs, resulting in substantial economic losses to the global swine industry [2]. Since 2011, PRV variant strains have emerged in China, leading to reduced protective efficacy of the widely used Bartha-K61 vaccine and posing new challenges for disease control [3]. In addition, PRV has been sporadically reported to cause viral encephalitis in humans following cross-species transmission, raising public health concerns [4,5]. As a large double-stranded DNA virus, the PRV genome is approximately 143 kb and encodes around 70 proteins [6]. Herpesvirus gene expression follows a strict temporal cascade: immediate-early (IE) genes are expressed first, encoding key regulatory proteins, followed by early (E) genes encoding DNA replication-associated enzymes, and finally late (L) genes encoding structural proteins [7]. The application of long-read sequencing technologies and multi-platform integration strategies has further revealed the complexity of the PRV transcriptome. These studies identified widespread transcript overlapping, nested structures, and 5′/3′ terminal variants, along with numerous previously unannotated non-coding RNAs. These findings indicate that PRV gene expression regulation is far more sophisticated than the classical three-stage cascade model suggests [8,9].

For herpes simplex virus type 1 (HSV-1), a closely related member of the Herpesviridae family, studies have shown that during the early phase of lytic infection, viral genomes rapidly associate with host histones to form nucleosome-like structures; however, this assembly is unstable and dynamic [10,11]. During lytic infection, ATRX can restrict chromatin accessibility of HSV-1 genomes associated with histone H3, and loss of ATRX significantly enhances transposase accessibility of viral DNA [12]. The composition, structure, and post-translational modifications of HSV-1 chromatin change continuously throughout the course of infection. These dynamic changes exert important influences on viral gene transcription profiles during lytic infection, latency, and reactivation, potentially determining the outcome of the lytic–latent balance [11]. However, knowledge regarding the chromatin dynamics of the PRV genome during infection and their impact on gene expression remains limited.

The Assay for Transposase-Accessible Chromatin using sequencing (ATAC-seq) is an efficient chromatin accessibility profiling technique that uses Tn5 transposase, which preferentially inserts into open chromatin regions, to assess chromatin state at the genome-wide level [13]. In recent years, ATAC-seq has been successfully applied to chromatin studies of multiple DNA viruses, including HSV-1 [14], human cytomegalovirus (HCMV) [15], and Epstein–Barr virus (EBV) [16], providing important tools for understanding the dynamic regulation of viral genome chromatin accessibility. In our previous study, we analyzed the chromatin accessibility and transcriptome of the host genome in PK15 cells following PRV infection using ATAC-seq and RNA-seq [17]. Based on this foundation, the present study further focuses on the viral genome by analyzing the chromatin accessibility state, transcriptional kinetics, and their associations with host gene expression at different post-infection time points. This work aims to provide new insights into the epigenetic regulatory mechanisms of PRV and virus–host interactions.

## 2. Results

### 2.1. Dynamic Changes in Viral ATAC-Seq Signals During PRV Infection

**Figure 1:**
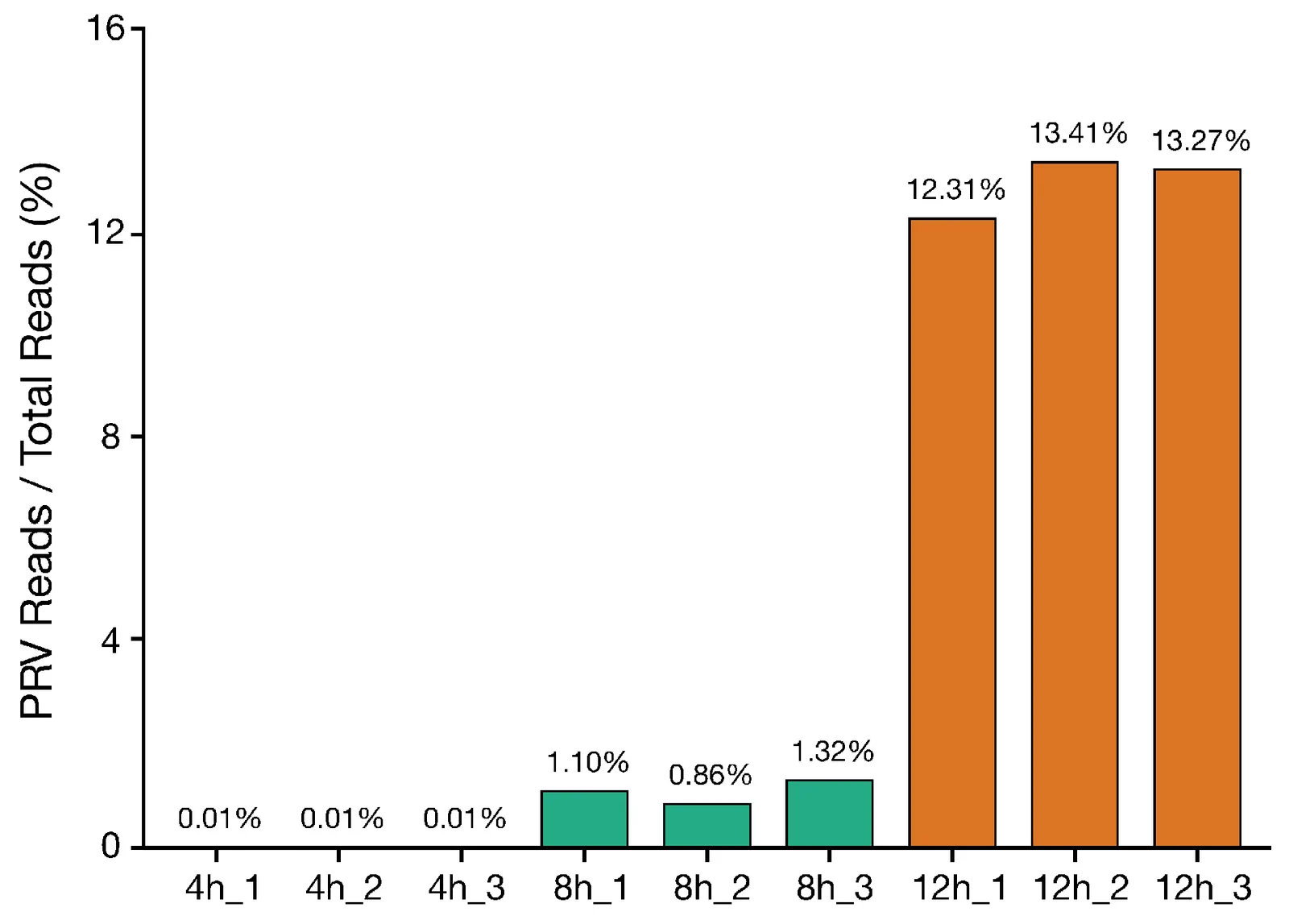
Proportion of PRV-mapped ATAC-seq reads at different time points post-infection.

To assess changes in chromatin accessibility of the PRV genome during infection, viral reads were extracted from ATAC-seq combined genome alignment data, and the proportion of viral reads at different time points was quantified. At 4 h post-infection (hpi), the proportion of viral reads was 0.01%. At 8 hpi, the proportion of viral reads rose to approximately 1%, with the three biological replicates reaching 1.10%, 0.86%, and 1.32%, respectively, indicating that viral DNA replication had been initiated. At 12 hpi, the proportion of viral reads rose further to 12.31%, 13.41%, and 13.27%, demonstrating rapid accumulation of large numbers of viral genomes in host cell nuclei while maintaining a highly accessible chromatin state (Figure 1). Good concordance was observed among the biological replicates at all three time points. The progressive rise in viral read proportion is consistent with the increasing viral titre, viral protein expression revealed by immunofluorescence and viral genome accumulation previously characterized for PRV in PK15 cells at the same post-infection time points [17,18].

### 2.2. Analysis of Chromatin Accessibility Across the PRV Genome

**Figure 2:**
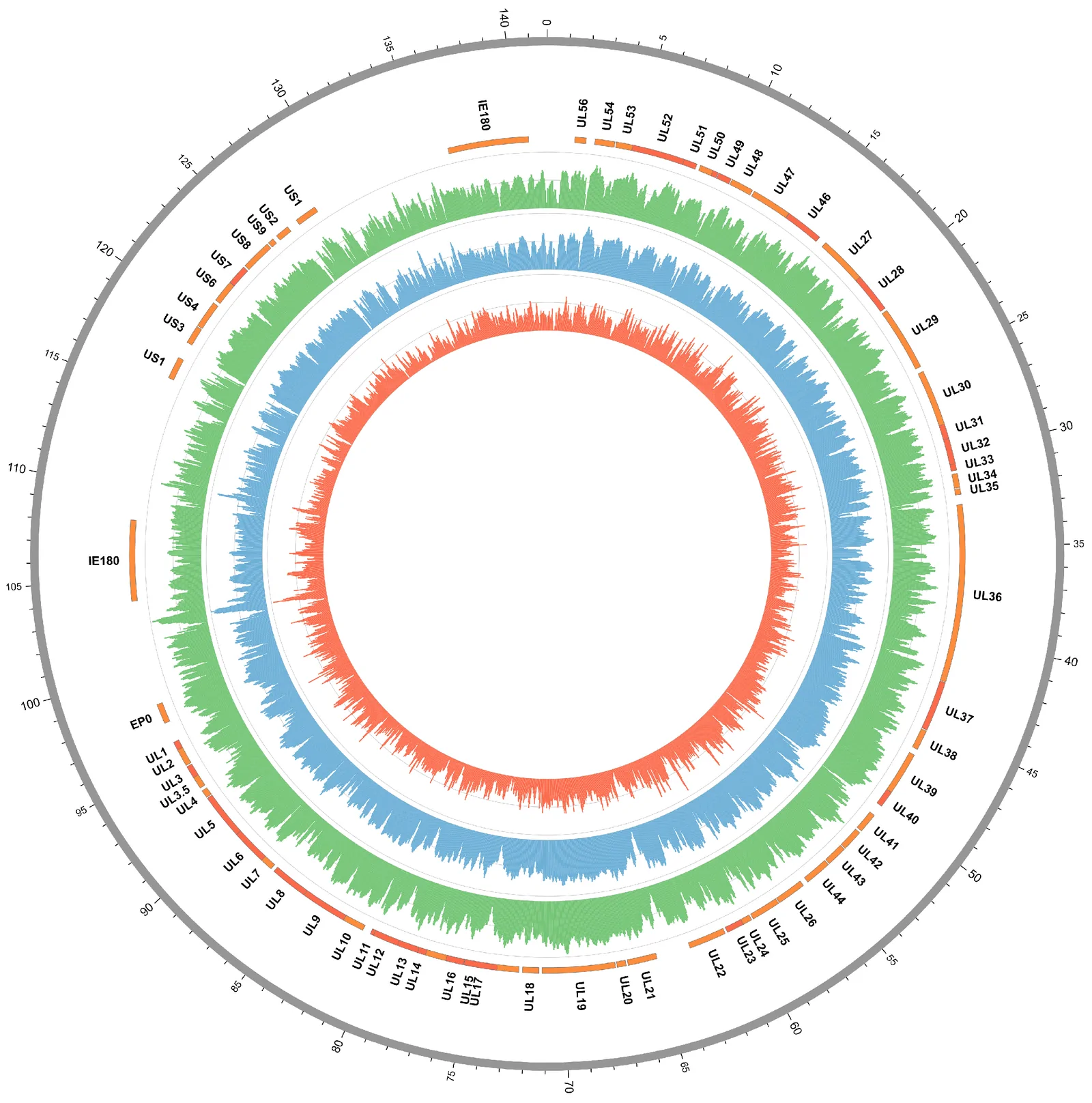
Circos plot of ATAC-seq signal distribution across the PRV genome. The outermost ring displays genomic coordinates and gene annotations. The three inner tracks represent ATAC-seq signals at 4 h (red), 8 h (blue), and 12 h (green) post-infection, each independently scaled to optimize visual contrast.

To characterize the chromatin accessibility landscape of the PRV genome, ATAC-seq data from all three time points were merged for peak calling analysis. A Circos plot was generated based on normalized BigWig signal files to visualize the distribution of accessible regions (Figure 2). The analysis identified 112 significant ATAC peaks distributed throughout the PRV genome. No apparent low-accessibility or closed chromatin regions were detected, as the peaks covered virtually all genomic regions. These results indicate that the PRV genome maintains a predominantly open and accessible chromatin state throughout lytic infection.

### 2.3. Fragment Length Analysis of the PRV Genome

**Figure 3:**
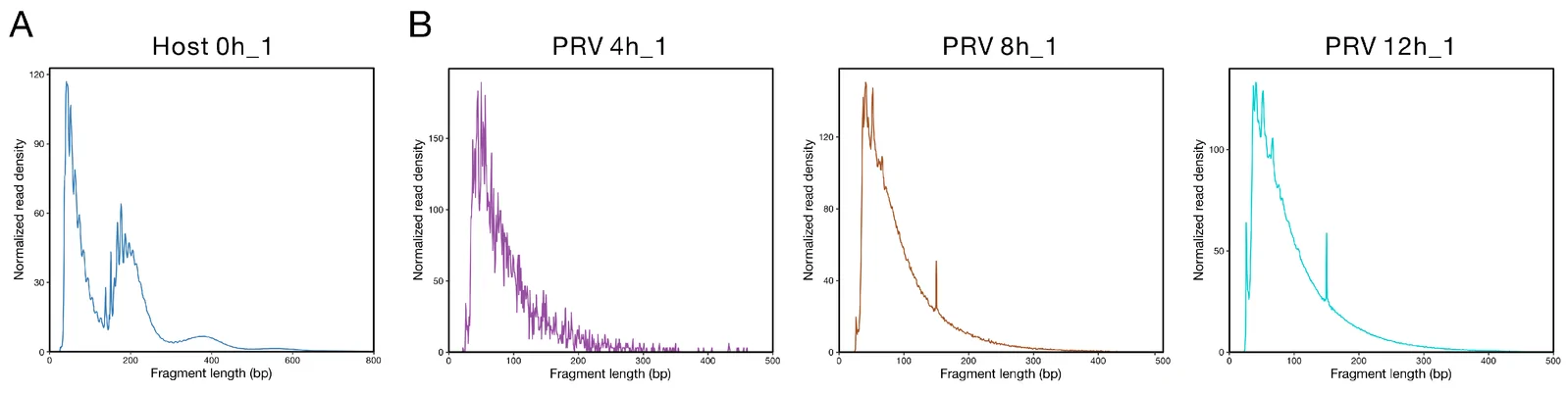
ATAC-seq fragment length distributions of the host and PRV genomes. (**A**) Host genome. (**B**) PRV genome at 4, 8 and 12 hpi.

In the typical host genome, ATAC-seq fragment lengths exhibit a pronounced nucleosome ladder pattern, with prominent peaks at approximately 200 bp (mono-nucleosome) and 400 bp (di-nucleosome) [19]. To determine whether the PRV genome adopts a similar chromatin organization, we analyzed the fragment length distributions of the host and PRV genomes. The host genome displayed the canonical nucleosome ladder (Figure 3A). In contrast, at all three time points, the fragment length distribution displayed a smooth, single-peak decay pattern, predominantly comprising short fragments within 100 bp (Figure 3B). No typical nucleosome ladder peaks were observed, indicating that the PRV genome does not form the stable, regularly phased nucleosome arrays characteristic of the host genome.

### 2.4. Principal Component Analysis of the PRV Transcriptome

**Figure 4:**
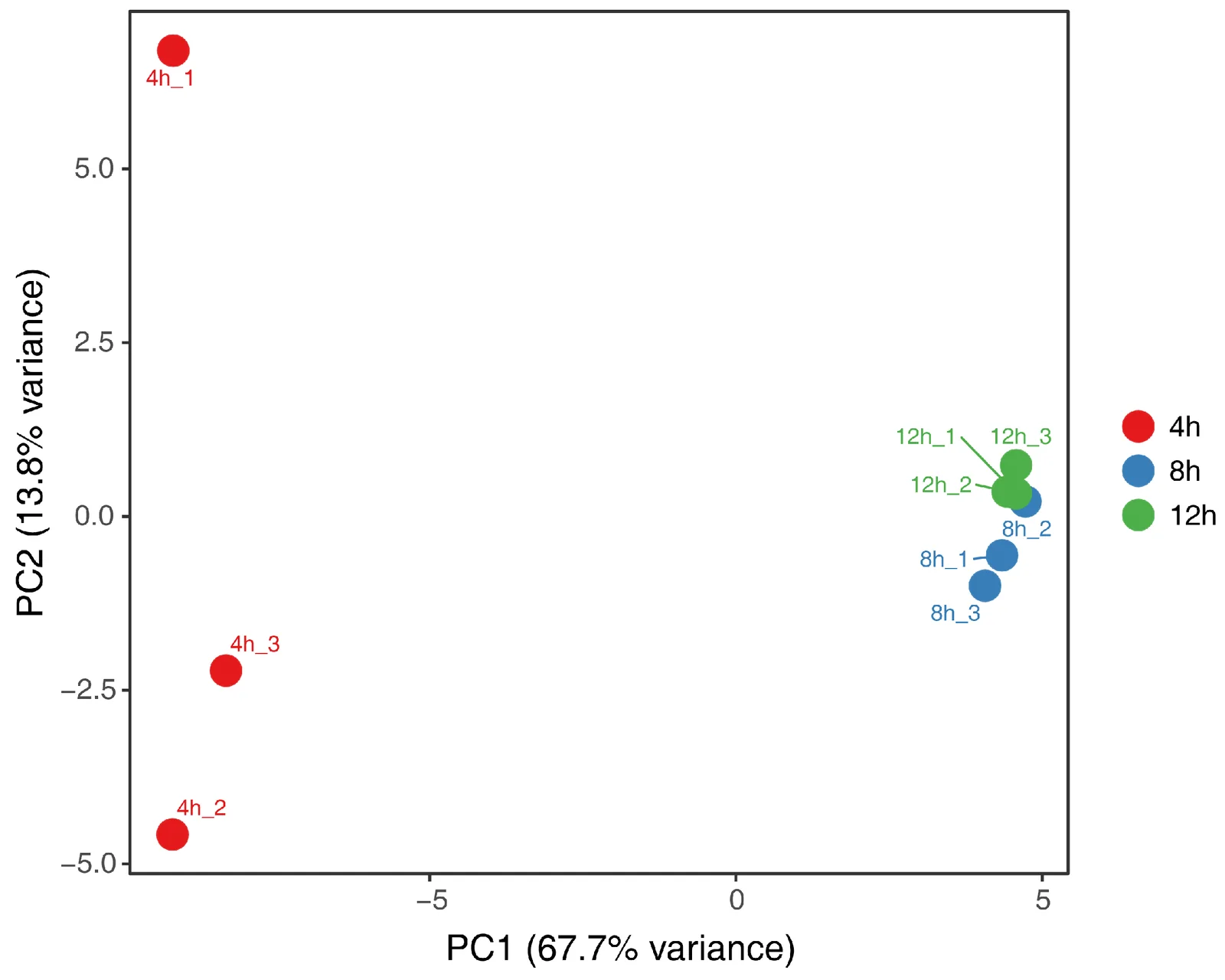
PCA of the PRV transcriptome.

Principal component analysis (PCA) was performed on the 66 expressed PRV genes. The results showed that PC1 and PC2 explained 67.7% and 13.8% of the total variance, respectively. The 4 hpi samples were located in the negative direction of PC1 with relatively greater dispersion among replicates, while the 8 hpi and 12 hpi samples clustered tightly in the positive direction of PC1 with only slight separation along PC2 (Figure 4). The greater dispersion among the 4 hpi replicates may be attributable to the very low viral read content at this early time point, which likely renders transcript quantification more susceptible to sampling noise. These results indicate significant time-dependent changes in the PRV transcriptome, with the 4-to-8 hpi interval representing the major transcriptome reprogramming phase.

### 2.5. Temporal Expression Profiles and Clustering Analysis of PRV Genes

**Figure 5:**
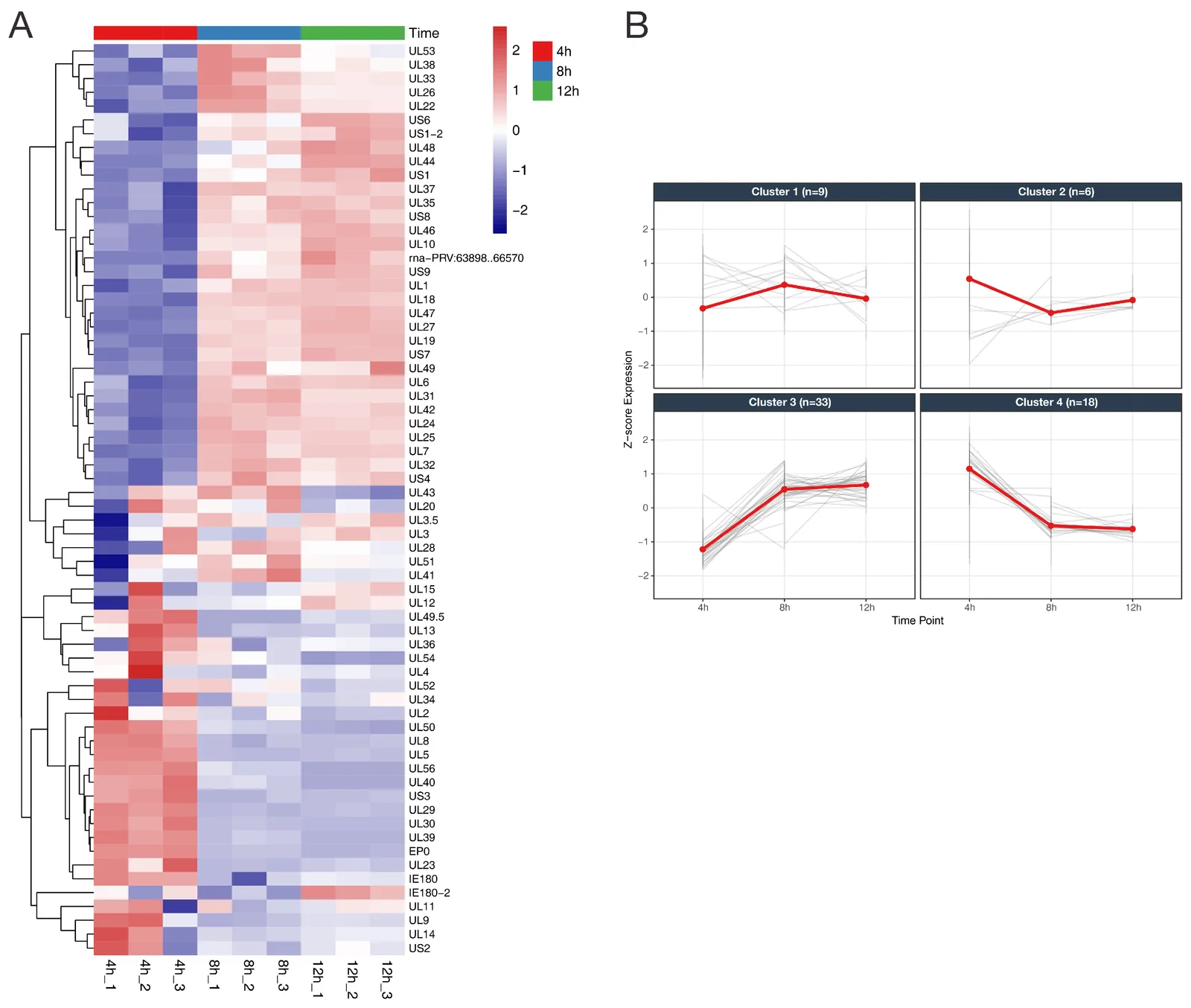
Temporal expression profiles and clustering of PRV genes. (**A**) Hierarchical clustering heatmap of expression profiles for 66 PRV genes. (**B**) K-means clustering (K = 4) of 66 PRV genes into four expression clusters. Grey lines show individual genes with three biological replicates per time point, and the red line shows the mean of all genes within the cluster.

A hierarchical clustering heatmap divided the 66 PRV genes into two groups: the upper gene group showed low expression at 4 hpi and high expression at 8/12 hpi, corresponding to late (L) genes predominantly encoding structural proteins (e.g., *UL53*, *UL48*, *UL44*, *UL46*, *UL27*, *UL22*, *US6*); the lower gene group showed the opposite trend, corresponding to immediate-early/early (IE/E) genes mainly involved in DNA replication and regulation (e.g., *IE180*, *EP0*, *UL29*, *UL30*, *UL39*, *UL40*, *UL23*) (Figure 5A).

K-means clustering (K = 4) partitioned the 66 expressed genes into four clusters representing distinct temporal expression patterns (Figure 5B, Table 1). Cluster 1 comprised 9 early (E) genes, including *UL51*, *UL28*, *UL36*, *UL41*, *UL43*, *UL20*, *UL12*, *UL3.5*, and *UL3*. These genes showed relatively low expression at 4 hpi, increased at 8 hpi, and then plateaued or slightly declined. This cluster contains genes involved in viral DNA packaging (*UL28*), tegument protein assembly (*UL36*, encoding the largest tegument protein), and host gene shutoff (*UL41*, encoding the virion host shutoff protein VHS), suggesting that these gene products begin to function in the middle phase of infection. Cluster 2 comprised 6 early-to-late (E-L) transitional genes, including *UL15*, *UL14*, *UL11*, *UL9*, *UL4*, and *US2*. Among these, *UL15* encodes the terminase and UL9 encodes the origin-binding protein; these gene products function continuously throughout infection. Cluster 3 was the largest cluster, comprising 33 late (L) genes, including *UL48*, *UL47*, *UL46*, *UL49*, *UL44*, *UL27*, *UL53*, *UL31*, *UL32*, *UL33*, *UL35*, *UL37*, *UL38*, *UL42*, *UL26*, *UL25*, *UL24*, *UL22*, *UL19*, *UL18*, *UL10*, *UL7*, *UL6*, *UL1*, *US1*, *US4*, *US6*, *US7*, *US8*, *US9*, *US1-2*, *IE180-2*, and *rna-PRV:63898..66570*. These genes showed low expression at 4 hpi and significantly increased at 8 and 12 hpi. This cluster encompasses a large number of genes encoding viral structural proteins, consistent with their late expression pattern meeting the demands of virion assembly. Cluster 4 comprised 18 immediate-early/early (IE/E) genes, including *IE180*, *EP0*, *UL54*, *UL52*, *UL50*, *UL49.5*, *UL29*, *UL30*, *UL34*, *UL39*, *UL40*, *UL23*, *UL13*, *UL8*, *UL5*, *UL2*, *US3* and *UL56*. These genes were most highly expressed at 4 hpi and subsequently declined significantly at 8 and 12 hpi. This cluster contains IE genes (*IE180*, *EP0*) and multiple DNA replication genes, and their high early expression reflects the molecular events of the infection initiation phase.

### 2.6. Expression Correlations Between Viral Genes and Host Inflammatory/Immune Genes

**Figure 6:**
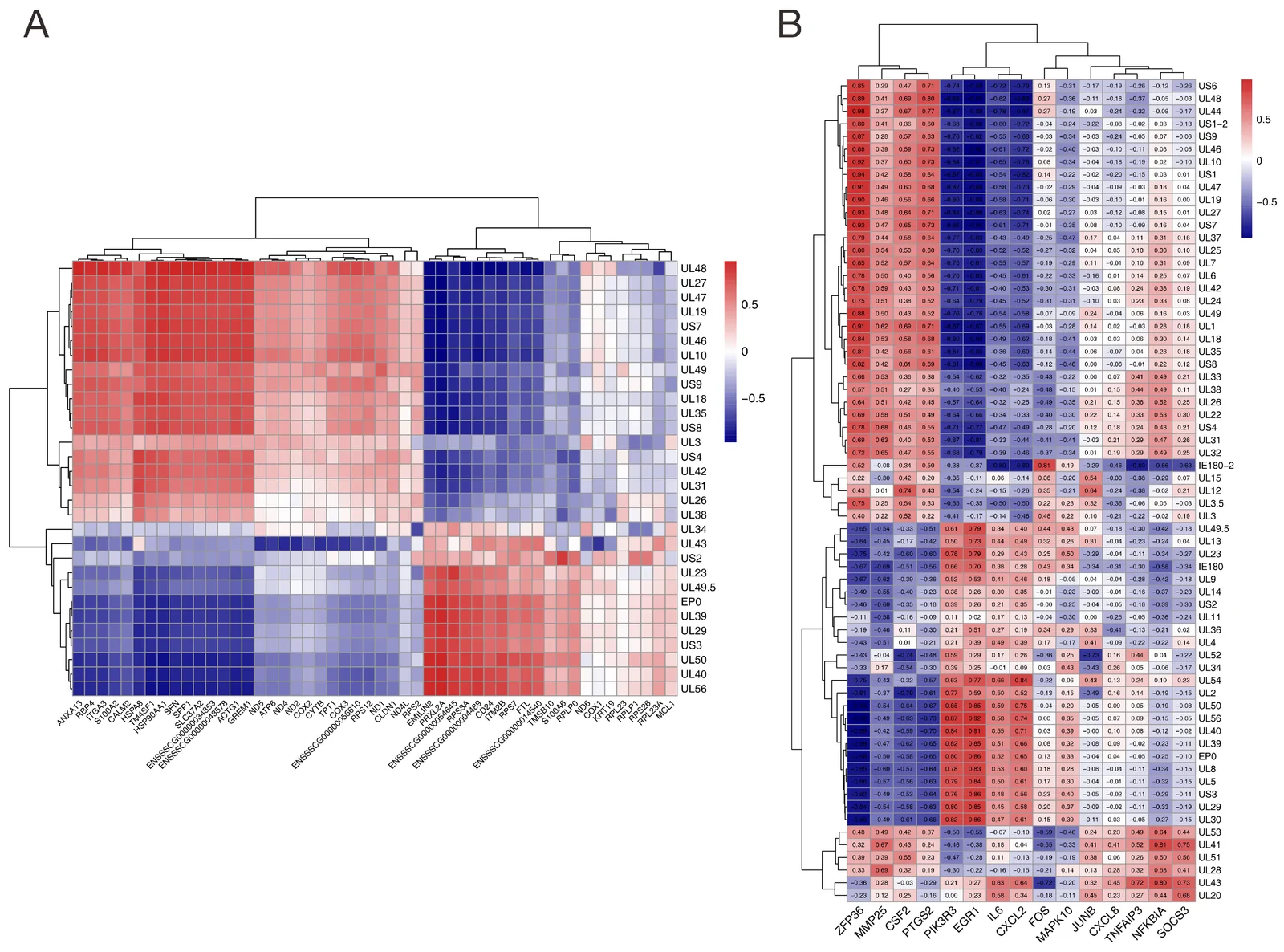
Expression correlation between PRV viral genes and host genes. (**A**) Pearson correlation heatmap of the 30 most highly expressed viral genes (rows) versus the 50 most variable host genes (columns). (**B**) Pearson correlation heatmap of all expressed PRV genes (rows) versus host inflammation- and immune-related genes (columns).

Because viral gene expression and host responses are tightly coupled during infection, we next examined how the temporal expression classes of viral genes relate to host gene expression, with particular attention given to inflammation- and immune-related genes. This analysis complements the host-centered analysis of our previous study [17] and aims to nominate candidate virus–host relationships. Hierarchical clustering was performed based on Pearson correlation coefficients between the top 30 highest-expressed viral genes and the top 50 most variable host genes. Late viral genes (*UL48*, *UL27*, *UL47*, *UL19*, *UL46*, *UL49*, etc.) showed strong positive correlations with host genes including *ANXA13*, *HSP90AA1*, *SPP1*, and *ACTG1*, while exhibiting strong negative correlations with *EMILIN2*, *PRXL2A*, and *CD24*. Early/immediate-early genes (*EP0*, *UL29*, *UL39*, *UL40*, *UL56*, *US3*, etc.) displayed opposite correlation patterns (Figure 6A). These results suggest that host gene expression undergoes systematic reprogramming as infection progresses.

Further analysis revealed distinct correlation patterns between viral genes and host inflammation/immune-related genes. Multiple late viral genes showed strong positive correlations with the inflammation-negative regulator *ZFP36*, while displaying significant negative correlations with the immediate-early transcription factor *EGR1*. In contrast, early genes *UL56* and *UL40* were positively correlated with *EGR1*. Similarly, *FOS* exhibited opposite correlation patterns with early and late viral genes, reflecting the important regulatory role of immediate-early response factors in the early phase of infection. Pro-inflammatory cytokines *IL6*, *CXCL2*, and *TNFAIP3* also showed differential correlations with viral genes from different temporal phases. Notably, *UL41* was positively correlated with the inflammation-negative feedback regulators *NFKBIA* and *SOCS3* (Figure 6B). These findings suggest that host immune responses are subject to multi-layered regulatory network modulation during PRV infection.

### 2.7. Integrated Analysis of ATAC-Seq and RNA-Seq

**Figure 7:**
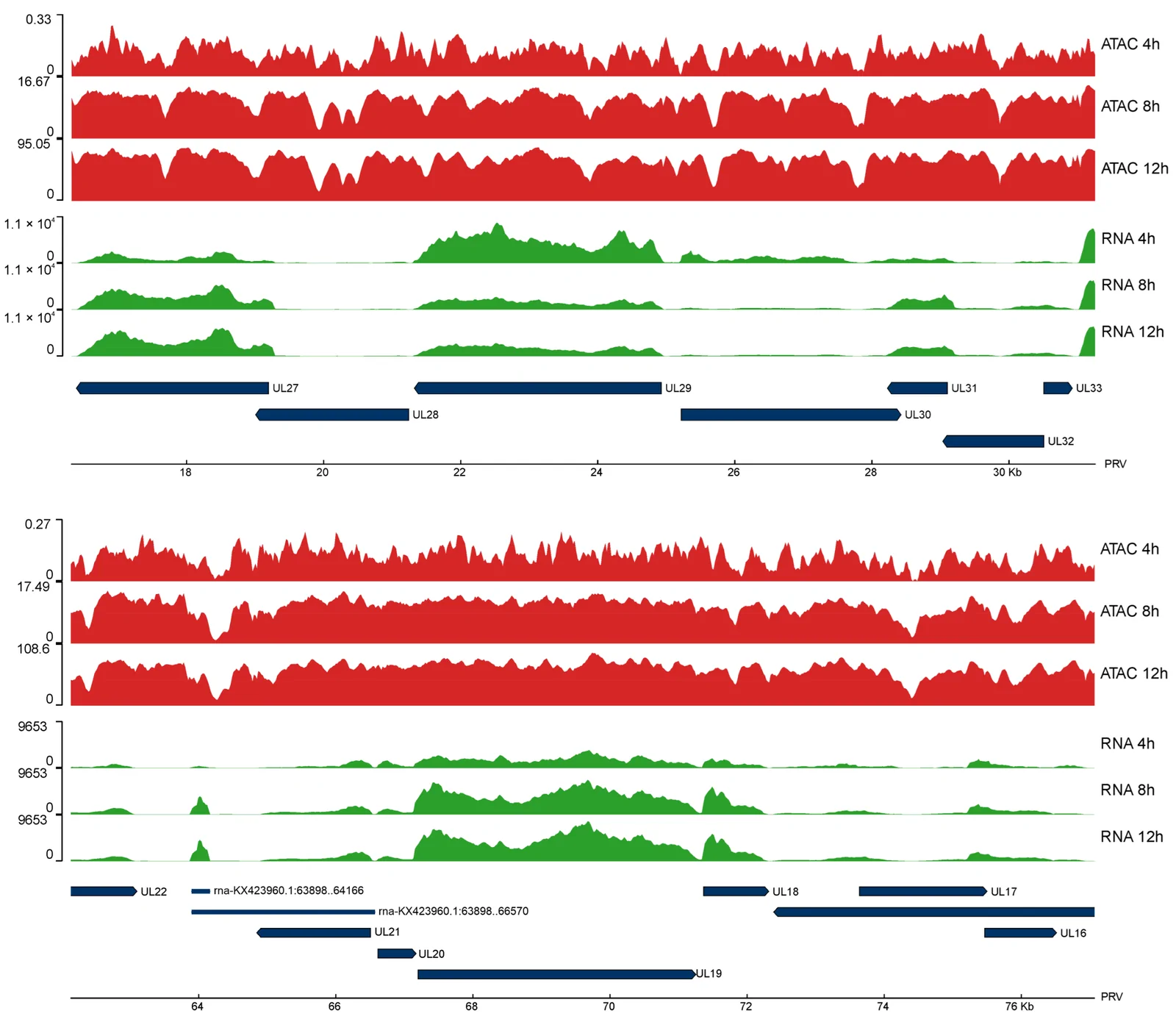
Integrated visualization of ATAC-seq and RNA-seq signals across representative PRV genomic regions.

To elucidate the correspondence between chromatin accessibility and transcriptional activity, we used pyGenomeTracks to display ATAC-seq and RNA-seq signal tracks for two representative regions of the PRV genome (Figure 7). In the *UL27*-*UL33* region, the overall ATAC-seq signal increased with the progression of infection, which mainly reflects the accumulation of viral genome copies during DNA replication rather than a locus-specific gain in chromatin accessibility. The RNA-seq signal displayed a clear temporal switch, with the early gene *UL29* showing a prominent transcription peak at 4 hpi that gradually diminished at 8 and 12 hpi, while the transcriptional signal of the late gene *UL27* synchronously increased, reflecting the transition from early-to-late expression. In the *UL16*-*UL22* region, ATAC-seq signals also increased over time, with the region encoding the major capsid protein VP5 (*UL19*) showing the highest ATAC-seq signal. The RNA-seq signal in this region was extremely low at 4 hpi but increased substantially at 8 and 12 hpi, indicating that the high transcriptional activity of late genes is closely associated with a highly open chromatin state.

## 3. Discussion

By integrating ATAC-seq and RNA-seq data, we characterized the chromatin accessibility dynamics and transcriptional regulatory features of the PRV genome during lytic infection of PK15 cells. Our results demonstrate that the PRV genome maintains a predominantly open and accessible chromatin state throughout lytic infection without regularly phased nucleosome positioning. Gene expression strictly follows the IE-E-L cascade pattern, accompanied by extensive temporal correlations between viral and host inflammation/immune gene expression. These findings provide a multi-dimensional epigenomic perspective for understanding the transcriptional regulation of PRV during lytic infection. The distribution of 112 ATAC peaks across the PRV genome and the absence of nucleosome ladder patterns in fragment length distributions are highly consistent with studies of HSV-1 lytic infection. Using ATAC-seq, it has been confirmed that despite being loaded with histone H3 early during HSV-1 infection, viral DNA remains highly accessible and does not form regularly arranged nucleosome structures. The host restriction factor ATRX can restrict DNA accessibility by altering viral chromatin structure [12]. The HSV-1 IE protein ICP0, corresponding to EP0 in PRV, maintains the open state of viral DNA by promoting histone removal and acetylation [20]. Meanwhile, ICP22 interferes with histone repositioning following transcription termination, resulting in aberrant open chromatin structures downstream of genes [21]. A recent study revealed that HSV-1 reshapes host chromatin architecture globally by hijacking RNA polymerase II (RNA Pol II) and TOP1, providing new mechanistic insights into chromatin dynamics during α-herpesvirus lytic infection [22]. Our study extends this understanding to PRV, suggesting that maintaining a predominantly open and accessible genome may be a common strategy of α-herpesviruses during lytic infection. It should be noted that ATAC-seq reports chromatin accessibility rather than histone occupancy. The absence of a nucleosome ladder therefore indicates the lack of regularly phased nucleosome arrays rather than a complete absence of histones, and definitive assessment of histone loading would require complementary approaches such as histone H3 chromatin immunoprecipitation [12]. The occasional regions of locally reduced ATAC signal are unlikely to represent genuine chromatin closure and may instead reflect insufficient sequencing coverage arising from the known bias against GC-rich fragments during PCR amplification and sequencing [23].

K-means clustering partitioned PRV genes into four expression clusters that represent the molecular basis of the IE-E-L cascade. Cluster 4 contains *IE180*, *EP0*, and numerous E genes that are highly expressed early and rapidly decline thereafter. Cluster 3, the largest gene cluster, encompasses nearly all structural protein-encoding L genes, with their substantial upregulation at 8 and 12 hpi consistent with the demands of virion assembly. PacBio long-read sequencing has revealed numerous polycistronic transcripts and splicing variants in the PRV transcriptome [8,9]. Furthermore, multiple studies have confirmed the high complexity of herpesvirus transcriptomes [24,25]. Future integration with long-read RNA-seq data is expected to provide deeper insights into the fine transcriptional regulatory networks of PRV genes. This temporally ordered three-class (IE-E-L) cascade is a hallmark of herpesvirus macromolecular synthesis [26].

Virus–host correlation analysis revealed a modular association architecture in which late and early viral genes formed positive- and negative-correlation modules with distinct host gene clusters, reflecting the systematic reprogramming of the host transcriptome over the course of infection. This observation is consistent with the findings of recent PRV transcriptomic studies [27,28]. At the level of inflammatory regulation, the strong positive correlation between *ZFP36* and multiple late viral genes is particularly noteworthy. *ZFP36* has been shown to promote the degradation of pro-inflammatory cytokine mRNAs, including those encoding tumor necrosis factor alpha (TNF-α) and interleukin-6 (IL-6), by recognizing AU-rich elements within the 3′UTR of target transcripts [29]. Its synchronous upregulation with late viral genes suggests that the host activates a negative feedback mechanism to restrain excessive inflammation during the late phase of infection. The pattern of *EGR1* being positively correlated with IE/E genes but negatively with L genes suggests its possible role as an important immediate-early response factor, consistent with the bidirectional regulatory role of *EGR1* in various viral infections [30]. Furthermore, the positive correlations of *UL41* with *NFKBIA* and *SOCS3* suggest that the VHS protein encoded by *UL41*, while mediating host mRNA shutoff, may indirectly modulate the negative feedback regulation of the nuclear factor kappa-light-chain-enhancer of activated B cells (NF-κB) and Janus kinase/signal transducers and activators of transcription (JAK/STAT) signaling pathways [31]. These virus–host associations are correlative and hypothesis-generating rather than evidence of direct regulation, and the candidate relationships identified here await future functional validation.

Integrated ATAC-seq and RNA-seq analysis revealed that certain PRV genomic regions retain a highly open chromatin state in the late phase of infection despite a marked decline in transcriptional output. This decoupling of chromatin accessibility from transcriptional activity indicates that temporal regulation of PRV gene expression is not governed by a simple chromatin switch. A likely contributor is VHS-mediated selective degradation of viral and host mRNAs, and PRV may additionally share mechanisms analogous to the competitive redistribution of RNA Pol II reported for HSV-1 [22,31]. Conversely, late-gene regions exhibited synchronous increases in both chromatin accessibility and transcriptional activity, suggesting that efficient late-gene transcription is contingent upon fully open chromatin. The 4, 8 and 12 hpi time points examined here were selected to capture the immediate-early, early, and late phases of a single PRV replication cycle and to match our previous study on the same infection model [17]. In herpesviruses, important chromatin events occur mainly within the first 3 h of infection [32]. Such early events were not resolved in the present study, and future work incorporating earlier time points will help to define the initial establishment of viral chromatin.

## 4. Materials and Methods

### 4.1. Cell Culture and Virus Infection

The detailed procedures for cell culture and virus infection are described in our previous publication [17]. Briefly, PK15 cells (Cat# GDC0061, China Center for Type Culture Collection, Wuhan, China) were cultured in minimum essential medium (MEM; Cat# BC-M-020, BioChannel Biotechnology, Nanjing, China) supplemented with 10% fetal bovine serum (FBS; Cat# A5256501, Gibco, Thermo Fisher Scientific, Waltham, MA, USA) and 1% non-essential amino acids (Cat# 11140050, Gibco, Thermo Fisher Scientific, Waltham, MA, USA) at 37 °C with 5% CO_2_. When cells reached 90% confluency, they were inoculated with PRV strain ZJ at a multiplicity of infection (MOI) of 0.1. After washing with phosphate-buffered saline (PBS; Cat# BC-BPBS-01, BioChannel Biotechnology), the cells were incubated for virus adsorption at 37 °C for 1 h, then the medium was replaced with MEM containing 2% FBS and incubation continued. Cell samples were collected at 0 h (mock-infected control), 4 h, 8 h, and 12 h post-infection, with three biological replicates per time point. The procedures for ATAC-seq and RNA-seq library construction and sequencing are detailed in our previously published article [17]. The raw ATAC-seq and RNA-seq data used in this study are available in the NCBI Sequence Read Archive database under BioProject accession number PRJNA1369774.

### 4.2. ATAC-Seq Data Processing and Viral Reads Extraction

Raw sequencing data were subjected to quality control, and reads were aligned to a combined reference genome consisting of the pig genome (Sus scrofa 11.1), the PRV genome (KX423960.1), and the porcine circovirus 1 genome (NC_001792.2) using Bowtie2 (v2.3.4.1). Reads mapping to the PRV chromosome were extracted from BAM files using samtools (v1.12), and the total aligned read counts and viral read counts for each sample were quantified using samtools idxstats to calculate the proportion of viral reads. 

### 4.3. ATAC-Seq Signal Normalization and Peak Calling

To eliminate differences in sequencing depth between samples at different time points, ATAC-seq signals were normalized to nuclear effective reads. For each sample, the number of nuclear effective reads was calculated as the total number of sequenced reads multiplied by the non-duplicate mapping rate and by the proportion of non-mitochondrial reads, so that PCR duplicates and mitochondrial reads were excluded. The nuclear effective reads of the three biological replicates of each time point were then summed, and a scale factor was obtained by dividing 1,000,000 by this sum. All signal tracks are therefore expressed per one million nuclear effective reads. BigWig files were generated using the bamCoverage tool in deepTools (v3.5.1) with the following parameters: --binSize 1, --smoothLength 10, --normalizeUsing None and --scaleFactor. These were set to the corresponding value for each time point. Based on normalized BigWig files, the Circos software (v0.69) was used to visualize the genome-wide ATAC signal distribution on the PRV genome. Because the number of viral reads at 4 hpi was too low for reliable independent peak calling and differed greatly from that at the later time points, the BAM files from the 4 h, 8 h and 12 h time points were merged into a master BAM file to define a consensus set of accessible regions using samtools merge. Peak calling was performed using MACS2 (v2.2.9.1) with the following parameters: -f BAM, -g 1.43e5, --nomodel, --shift -100, --extsize 200, --keep-dup all, and -q 0.01.

### 4.4. RNA-Seq Data Processing and Quantification

RNA-seq data were also aligned using the combined genome alignment strategy. PRV reads were extracted from aligned BAM files using samtools, and counts per million (CPM)-normalized BigWig files were generated using deepTools bamCoverage. Transcript-level quantification of PRV genes was performed using featureCounts (v2.0.3; Subread package). To eliminate the effects of gene length and sequencing depth differences, raw counts were converted to transcripts per million (TPM) values.

### 4.5. Principal Component Analysis (PCA) and Clustering Analysis

PCA was performed on log_2_(TPM + 1)-transformed expression matrices. Genes with zero variance across all samples were removed prior to analysis. The R (v4.5.1) function prcomp was used for computation with data centering and scaling. PCA plots were generated using the ggplot2 package (v4.0.0). Gene expression heatmaps were constructed using the pheatmap package (v1.0.13). Low-expression genes were first filtered (total TPM > 10), and the filtered expression matrix was subjected to row normalization (Z-score). Samples were arranged in chronological order and genes (rows) were subjected to hierarchical clustering. The number of K-means clusters was set to four to reflect the canonical cascade of herpesvirus gene expression [26], with the late genes further resolved into a transitional and a strictly late group, giving the IE/E, E, E-L and L kinetic classes.

### 4.6. Virus–Host Gene Correlation Analysis

The top 30 viral genes with the highest mean TPM values were selected, along with the top 50 host genes with the largest expression variance (filter criterion: expression > 1 TPM in at least half of all samples). Pearson correlation coefficient matrices between viral and host genes were computed, and correlation heatmaps were generated using pheatmap. Gene pairs with |*r*| > 0.8 were selected for in-depth analysis. Host genes related to inflammation/immune responses were further selected, and their Pearson correlation coefficients with all expressed PRV genes were calculated to generate correlation heatmaps. ATAC-seq and RNA-seq BigWig signal tracks were loaded using pyGenomeTracks (v3.9) for visualization of representative genomic regions.

## 5. Conclusions

By integrating ATAC-seq and RNA-seq analyses, this study characterizes the chromatin accessibility dynamics and transcriptional regulatory landscape of the PRV genome during lytic infection of PK15 cells. The PRV genome maintains a predominantly open and accessible chromatin state throughout lytic infection without regularly phased nucleosome positioning. Gene expression adheres strictly to the IE-E-L temporal cascade and exhibits extensive correlations with host inflammatory and immune gene expression. These findings provide a valuable reference for understanding the epigenetic regulation of α-herpesviruses and the mechanisms governing virus–host interactions.

## Figures and Tables

**Table 1:** K-means clustering of PRV gene expression patterns.

Cluster	Expression Pattern	Gene Count	Gene List
Cluster 1 (E)	Early (4 h → 8 h increase, then stable)	9	*UL51*, *UL28*, *UL36*, *UL41*, *UL43*, *UL20*, *UL12*, *UL3.5*, *UL3*
Cluster 2 (E-L)	Early–late transition (relatively stable)	6	*UL15*, *UL14*, *UL11*, *UL9*, *UL4*, *US2*
Cluster 3 (L)	Late (4 h low → 8 h/12 h increase)	33	*UL48*, *UL47*, *UL46*, *UL49*, *UL44*, *UL27*, *UL53*, *UL31*, *UL32*, *UL33*, *UL35*, *UL37*, *UL38*, *UL42*, *UL26*, *UL25*, *UL24*, *UL22*, *UL19*, *UL18*, *UL10*, *UL7*, *UL6*, *UL1*, *US1*, *US4*, *US6*, *US7*, *US8*, *US9*, *US1-2*, *IE180-2*, *rna-PRV:63898..66570*
Cluster 4 (IE/E)	Immediate-early/Early (4 h high → 8 h/12 h decrease)	18	*IE180*, *EP0*, *UL54*, *UL52*, *UL50*, *UL49.5*, *UL29*, *UL30*, *UL34*, *UL39*, *UL40*, *UL23*, *UL13*, *UL8*, *UL5*, *UL2*, *US3*, *UL56*

## Data Availability

The original contributions presented in this study are included in the article. Further inquiries can be directed to the corresponding author.

## Abbreviations

The following abbreviations are used in this manuscript:

3′UTR	3′ Untranslated Region
ATAC-seq	Assay for Transposase-Accessible Chromatin using Sequencing
CPM	Counts Per Million
E	Early
E-L	Early-to-Late
EBV	Epstein–Barr Virus
FBS	Fetal Bovine Serum
HCMV	Human Cytomegalovirus
hpi	Hours Post-Infection
HSV-1	Herpes Simplex Virus Type 1
IE	Immediate-Early
IL-6	Interleukin-6
JAK/STAT	Janus Kinase/Signal Transducers and Activators of Transcription
L	Late
MACS2	Model-Based Analysis of ChIP-Seq 2
MEM	Minimum Essential Medium
MOI	Multiplicity of Infection
mRNA	Messenger Ribonucleic Acid
NF-κB	Nuclear Factor Kappa-Light-Chain-Enhancer of Activated B Cells
PBS	Phosphate-Buffered Saline
PCA	Principal Component Analysis
PRV	Pseudorabies Virus
RNA Pol II	RNA Polymerase II
RNA-seq	RNA Sequencing
SuHV-1	Suid Herpesvirus 1
TNF-α	Tumor Necrosis Factor Alpha
TPM	Transcripts Per Million
VHS	Virion Host Shutoff
